# A Missense Mutation in* POU4F3* Causes Midfrequency Hearing Loss in a Chinese ADNSHL Family

**DOI:** 10.1155/2018/5370802

**Published:** 2018-04-04

**Authors:** Xue Gao, Jin-Cao Xu, Wei-Qian Wang, Yong-Yi Yuan, Dan Bai, Sha-Sha Huang, Guo-Jian Wang, Yu Su, Jia Li, Dong-Yang Kang, Mei-Guang Zhang, Xi Lin, Pu Dai

**Affiliations:** ^1^Department of Otolaryngology, Head and Neck Surgery, Chinese PLA General Hospital, No. 28 Fuxing Road, Beijing 100853, China; ^2^Department of Otolaryngology, The General Hospital of the PLA Rocket Force, No. 16 XinWai Da Jie, Beijing 100088, China; ^3^Department of Otolaryngology, Head and Neck Surgery, School of Clinical Medicine, Xi'an Medical University, Xin Wang Road No. 1, Xi'an 710041, China; ^4^Department of Otolaryngology, Emory University School of Medicine, 615 Michael Street, Whitehead Biomedical Research Bldg, Rm No. 543, Atlanta, GA 30322, USA

## Abstract

Hereditary nonsyndromic hearing loss is extremely heterogeneous. Mutations in the POU class 4 transcription factor 3* (POU4F3)* are known to cause autosomal dominant nonsyndromic hearing loss linked to the loci of DFNA15. In this study, we describe a pathogenic missense mutation in* POU4F3* in a four-generation Chinese family (6126) with midfrequency, progressive, and postlingual autosomal dominant nonsyndromic hearing loss (ADNSHL). By combining targeted capture of 129 known deafness genes, next-generation sequencing, and bioinformatic analysis, we identified* POU4F3* c.602T>C (p.Leu201Pro) as the disease-causing variant. This variant cosegregated with hearing loss in other family members but was not detected in 580 normal controls or the ExAC database and could be classified as a “pathogenic variant” according to the American College of Medical Genetics and Genomics guidelines. We conclude that* POU4F3* c.602T>C (p.Leu201Pro) is related to midfrequency hearing loss in this family. Routine examination of* POU4F3 *is necessary for the genetic diagnosis of midfrequency hearing loss.

## 1. Introduction

Hearing loss is a common sensory defect in humans. Nonsyndromic hereditary forms, in which hearing loss is the only clinical sign, are known to be genetically heterogeneous. So far, more than 30 genes responsible for autosomal dominant nonsyndromic hearing loss (ADNSHL) have been identified (Hereditary Hearing Loss homepage, http://hereditaryhearingloss.org). Most patients with ADNSHL show large variations in age of onset and degrees of variety. According to the affected frequency, the phenotypes are divided into low-frequency, midfrequency, high-frequency, and all-frequency hearing loss. Midfrequency hearing loss (i.e., a U-shape audiogram) is a rare form of hearing loss, and six associated genes have been reported to date:* EYA4, TECTA, COL11A2, CCDC50, POU4F3, *and* SLC44A4 *[1–6]. Thus, in clinical molecular diagnosis, the hearing loss phenotype in a patient can aid the selection of a limited number of genes for mutational analysis.

This study identified one Chinese family with ADNSHL (number 6126), in which affected individuals showed typical midfrequency hearing loss. Using next-generation sequencing, we performed large-scale mutational screening of 129 known deafness-related genes and identified one novel dominant disease-segregating mutation, c.602T>C (p.Leu201Pro) in the* POU4F3 *gene, as the causative mutation that led to the midfrequency hearing loss phenotype in this family.

## 2. Materials and Methods

### 2.1. Clinical Data

Family 6126 is a four-generation Chinese family with autosomal dominant, late onset, progressive, nonsyndromic sensorineural hearing loss. Fully informed written consent was attained from each subject or their guardians. The study was approved by the Chinese PLA General Hospital's ethics research committees. Clinical information was gathered through multiple interviews with all participating members of the family. Medical history collection, otoscopy, physical examination, pure tone audiometric examination, and vestibular function were performed as previously described [7]. CT scans of the temporal bone in the index patients were performed.

### 2.2. DNA Preparation

Genomic DNA was extracted from peripheral blood using a blood DNA extraction kit according to the manufacturer's instructions (TianGen, Beijing, China).

### 2.3. Deafness Gene Capture and Illumina Library Preparation

Three prevalent deafness-associated genes,* GJB2*,* SLC26A4*, and mtDNA*12SrRNA*, were first screened for mutations in all participating cases and controls. Two affected individuals (II:1 and III:1) and one unaffected individual (II:3) of family 6126 were subjected to a gene panel containing 129 deafness genes. Capture and NGS of the coding exons for the 129 deafness genes and their flanking 100 bps (Supplemental Table 1) were performed on an Illumina HiSeq 2000 by Otogenetics Corporation (Norcross, GA, USA).

The details of the deafness gene capture, sequencing, and bioinformatics analysis methods have been described in detail previously [8]. According to the autosomal dominant pattern of inheritance, only variants that were heterozygous in the affected siblings were selected as candidates.

Segregation of the* POU4F3 c.602T>C (p.Leu201Pro)* variant was tested in seven family members (II:1, II:3, II:4, II:5, III:1, III:2, and IV:1), including the three whose gDNA had been subjected to NGS screening, using polymerase chain reaction (PCR) (primer sequences available on request) followed by bidirectional Sanger sequencing. Sequence alterations were identified by alignment with the* POU4F3 *GenBank sequence (NM_002700.2 and NP_002691.1) using the GeneTools software. To identify pathogenic mutations, cosegregation analysis was performed with the family members and an in-house database of 481 Chinese controls with normal hearing.

### 2.4. Multiple Sequence Alignment

Multiple sequence alignment was performed according to a Homologene program with default settings and the sequences* NP_002691.1 (H. sapiens)*,* XP_001100319.1 (M. mulatta)*,* XP_527063.1 (P. troglodytes)*,* XP_544328.1 (C. lupus)*,* NP_001178964.1 (B. taurus)*,* NP_620395.2 (M. musculus)*,* NP_001102359.1 (R. norvegicus)*,* NP_990090.1 (G. gallus)*,* NP_571353.1 (D. rerio)*,* NP_524876.1 (D. melanogaster)*,* XP_308015.5 (A. gambiae)*,* and XP_002935313.1 (X. tropicalis)* (https://www.ncbi.nlm.nih.gov/homologene?cmd=Retrieve&dopt=MultipleAlignment&list_uids=2023).

## 3. Results

### 3.1. Family and Clinical Evaluations

**Figure 1:**
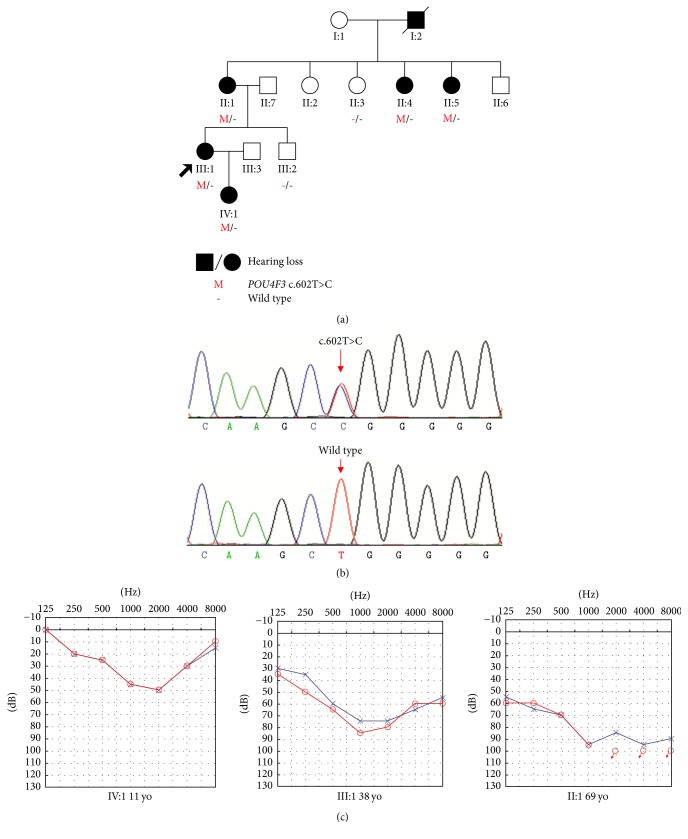
*Pedigree, mutational analysis, and audiogram of Chinese family 6126 with ADNSHI. *(a) The proband is indicated by an arrow. Subjects II:1, II:3 and III:1 were tested by NGS. (b) DNA sequencing profile showing the* POU4F3 *c.602T>C cosegregated with the hearing loss. (c) Audiogram showed bilateral sensorineural hearing impairment of affected subjects II:1, III:1, and IV:1 (red: right ear; blue: left ear).

Family 6126 is a four-generation Chinese family with ADNSHL (Figure 1) and includes six affected patients: II:1 (female, 69 years old), II:4 (female, 60 years old), II:5 (female, 57 years old), III:1 (female, 42 years old), and IV:1 (female, 11 years old). For this family, hearing impairment was postlingual, late onset (after 10 years old), and progressive. Their hearing loss progressed gradually with advancing age. Audiograms show that although low-frequency and high-frequency hearing were normal in the beginning, their hearing would ultimately deteriorate at all frequencies. Flatter audiogram configurations were observed at 69 years of age (II:1), whereas the audiograms of III:1 and IV:1 were U-shaped (Figure 1(c)). For affected subjects II:4 and II:5, audiograms were unavailable.

Detailed vestibular analysis was performed in III:1, who did not complain about dizziness, vertigo, or imbalance. Vestibular tests revealed normal vestibular function via caloric tests. All position tests produced no nystagmus without vertigo sensation. Affected individuals did not have obvious delayed gross motor development. The physical examinations of all participating members revealed no signs of systemic illness or dysmorphic features. High-resolution computed tomography of the temporal bone in III:1 was normal, excluding inner ear malformations. This phenotype was consistent with that reported for DFNA15.

### 3.2. Mutation Detection and Analysis

**Figure 2:**
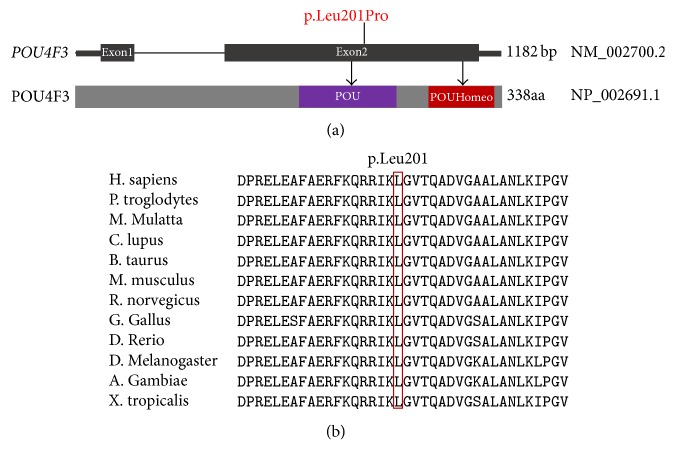
*Conservation analysis and genomic structure of POU4F3 based on the open reading frame (NM_002700.2) containing 2 exons (black rectangles)*. (a) The position of* POU4F3* c.602T>C (p.Leu201Pro) is highlighted in red and shown both at the gene (top) and the protein level (bottom). The protein diagram depicts the predicted functional domains and sequence motifs. (b) Protein alignment showing POU4F3 p.Leu201 occurred at evolutionarily conserved amino acids (in red box) across twelve species.

We sequenced all the coding exons plus ~100 bp of the flanking intronic sequence of 129 deafness genes in one unaffected (II:4) individual and two affected (II:1 and III:1) individuals of family 6126. One variant leading to amino acid change was detected in* POU4F3*: c.602T>C (p.Leu201Pro), which is located within exon 2 (Figure 2(a)). This variant has not been reported previously nor found in the ExAC database (http://exac.broadinstitute.org/) and was not observed in the 481 Chinese controls with normal hearing. The substitution occurred in an evolutionarily conserved region across different species in the POU domain (Figure 2(b)) and is predicted to be damaging by SIFT, Polyphen2, and CADD.

Using Sanger sequencing, seven participating family members (five affected and two unaffected) in family 6126 were genotyped to identify the mutation.* POU4F3 *heterozygous variant p.Leu201Pro was found in five patients (Figure 1), consistent with autosomal dominant inheritance.

These data, together with the clinical presentation of the affected siblings and consistent autosomal dominant inheritance of the mutations in the affected and unaffected members, indicate that* POU4F3* c.602T>C (p.Leu201Pro) is the cause of hearing impairment in this family.

## 4. Discussion

Inner ear hair cells play a crucial role in the mechanical transmission of sound and stimulation of the auditory nerve. A defect in hair cells in the cochlea can be a major reason for sensorineural hearing loss. POU4F3, a POU domain class IV protein, has two exons and encodes a protein of 338 amino acids that belongs to the POU domain family of transcription factors, which are expressed specifically in inner ear hair cells and play a critical role in the maturation, differentiation, and maintenance of inner ear hair cells [9]. POU4F3 contains two conserved DNA-binding domains (a POU-specific domain and a POU homeodomain; amino acids 179–256 and 274–333, resp.), which are the main functional parts [10].

In 1998,* POU4F3 *was first described as a disease-causing gene within the DFNA15 locus in an Israeli Jewish family [11]. So far, 27 variants in* POU4F3* (13 missense variants, 8 frameshift variants, 4 nonsense variants, and 1 splice-site variant) (Table 1) and a whole deletion of POU4F3 [12] have been reported to cause ADNSHL with variable ages of onset and degrees of severity in various ethnic groups, including Chinese, Japanese, Dutch, Korean, and Brazilian populations [6, 10, 13–18]. Recently, Kitano et al. reported that* POU4F3* variants represent the third largest cause of ADNSHL (2.5%, 15/602) in Japan and usually presented with mid- or high-frequency hearing loss. They also noticed that patients with truncating variants showed earlier onset and slower progression of hearing loss compared to those with nontruncating variants [6]. Through next-generation sequencing, He et al. reported that mutation in* POU4F3* is a relatively common (3/18) cause of ADNSHL among Han Chinese people. Notably, most causative variants were located within or close to the POU-specific domain or the POU homeodomain, the two conserved DNA-binding domains of POU4F3 encoded in exon 2. The age of onset of hearing loss, ranging from the first to fourth decade of life, differs among* POU4F3 *mutations. In the present study, the earliest age of onset of hearing loss for affected family members was recorded at 11 years old (IV:1). Although the pathogenic mechanisms underlying hearing impairment of patients with* POU4F3* variants remain unclear, the mechanism of haploinsufficiency has been supported by several studies [12, 19, 20].

The missense mutation p.Leu201Pro is located within the POU domain and encodes a proline at position 201 instead of the highly conserved leucine, which is close to the two reported dominant mutations of* POU4F3* (c.603-604delGG [p.Leu201fs∗12] and c.602delT [p.Leu201fs]) [21, 22]. We speculate that to some extent, this area of the genome is unstable and susceptible to mutation.

In this study, we identified a novel missense mutation, c.602T>C in* POU4F3*, in one Chinese family (6216) with ADNSHL. Younger patients in this family demonstrated midfrequency hearing loss with no additional clinical symptoms. Our results strongly suggest that this missense mutation is related to hearing loss in this family, which has a putative autosomal dominant inheritance pattern.

## 5. Conclusions

In summary, we describe the clinical and genetic characteristics of a Chinese family (number 6126) with postlingual ADNSHL caused by* POU4F3* c. 602 T>C (p. Leu201Pro) through multiple deafness gene capture and next-generation sequencing. Notably, the hearing impairment of affected individuals in this family is mainly midfrequency. This specific audiogram should be considered in clinical genetic diagnosis and counselling. Therefore, screening for* POU4F3* in ADNSHL patients with a postlingual, progressive, and U-shape audiogram is necessary for efficient genetic diagnosis and intervention.

## Figures and Tables

**Table 1:** Overview of *POU4F3* mutations described in DFNA15.

Number	Nucleotide change	Protein change	Exon	Domain	Origin	Audiometric configuration	Reference
(1)	Whole deletion of *POU4F3 *				Brazil	Flat and HF	Freitas et al., 2014
(2)	c.74dupA	p.His25fs*∗*18	1		Japan	HF	Kitano et al., 2017
(3)	c.120+1G>C		1		China	Flat	He et al., 2016
(4)	c.191A>T	p.Asp64Val	2		Japan	HF	Kitano et al., 2017
(5)	c.337C>T	p.Gln113Ter	2		China		Zhang et al., 2016
(6)	c.367delA	p.Ile123fs*∗*3	2		Japan	MF	Kitano et al., 2017
(7)	c.427C>T	p.Gln143Ter	2		Japan	MF	Kitano et al., 2017
(8)	c.491C>G	p.Pro164Arg	2	POU	China	Flat and HF	Wei et al., 2014
(9)	c.574G>T	p.Glu192Ter	2	POU	Japan	HF	Kitano et al., 2017
(10)	c.581T>A	p.Phe194Tyr	2	POU	Japan	HF	Kitano et al., 2017
(11)	c.602T>C	p.Leu201Pro	2	POU	China	MF	This study
(12)	c.602delT	p.Leu201fs*∗*3	2	POU	China	HF	Cai et al., 2016
(13)	c.603_604delGG 2	p.Val203Aspfs*∗*11	2	POU	China	N/A	Yang et al., 2013
(14)	c.662_675del14	p.Gly221Glufs*∗*14	2	POU	Korea	HF	Lee et al., 2010
(15)	c.665C>T	p.Ser222Leu	2	POU	Japan	HF	Kitano et al., 2017
(16)	c.668T>C	p.Leu223Pro	2	POU	The Netherlands	Flat, MF, and HF	Collin et al., 2008
(17)	c.680delC	p.Thr227fs*∗*13	2	POU	Japan	MF	Kitano et al., 2017
(18)	c.694G>A	p.Glu232Lys	2	POU	Korea	HF	Baek et al., 2012
(19)	c.718A>T	p.Asn240Tyr	2	POU	Japan	MF	Kitano et al., 2017
(20)	c.841A>G	p.Ile281Val	2	POU Homeobox	Japan	HF	Kitano et al., 2017
(21)	c.865C>T	p.Leu289Phe	2	POU Homeobox	The Netherlands	Flat, MF, and HF	Collin et al., 2008
(22)	c.884_891del8	Ile295Thrfs*∗*5	2	POU Homeobox	Israel	HF	Vahava et al., 1998
(23)	c.896C>T	p.Pro299Leu	2	POU Homeobox	Japan	MF	Kitano et al., 2017
(24)	c.932T>C	p.Leu311Pro	2	POU Homeobox	China	HF	He et al., 2016
(25)	c.976A>T	p.Arg326Ter	2	POU Homeobox	Japan	HF	Kitano et al., 2017
(26)	c.977G>A	p.Arg326Lys	2	POU Homeobox	Korea	HF	Kim et al., 2013
(27)	c.982A>G	p.Lys328Glu	2	POU Homeobox	Taiwan	HF	Lin et al., 2017
(28)	c.1007delC	p.Ala336fs	2	POU Homeobox	Japan	N/A	Mutai et al., 2013

*Abbreviations*. HF: high-frequency hearing loss; MF: midfrequency hearing loss.

## Supplementary Materials

Supplementary Materials Supplemental Table 1: gene panel containing 129 deafness genes.
