# Supplementary material for: A Missense Mutation in POU4F3 Causes Midfrequency Hearing Loss in a Chinese ADNSHL Family
Source: Biomed Res Int. 2018 Apr 4;2018:5370802. doi: 10.1155/2018/5370802 (PMC5904794; doi:10.1155/2018/5370802)
Supplement: Supplementary Materials — Supplemental Table 1: gene panel containing 129 deafness genes. [file 5370802.f1.pdf]

Supplemental table 1

129 deafness genes

ACTB	FGF3	MYO15A	SPINK5	MTTS2	MSRB3
ACTG1	GATA3	MYO1A	STRC	MTTL2	NDP
ATP6V1B1	GJA1	MYO1C	TBL1X	FGFR3	SNAI2
BCS1L	Gjb1	MYO1F	TCF21	mir96	SERPINB6
BSND	GJB2	MYO3A	TECTA	mir182	SIX1
CATSPER2	GJB3	MYO6	TFCP2	mir183	SIX5
CCDC50	GJB4	MYO7A	TIMM8A	GRXCR1	TPRN
CDH23	GJB6	NR2F1	TMC1	USH2C	FAS
CLDN14	GRHL2	OTOA	TMIE	USH2A	EDNRA
COCH	GSTP1	OTOF	TMPRSS3	USH1G	EDNRB
COL11A2	JAG1	OTOR	TMPRSS5	USH3A	MITF
COL9A3	KCNE1	PAX3	TRIOBP	HGF	ECE1
CRYM	KCNJ10	PCDH15	USH1C-a-b	TJP2	FKH10
DFNA5	KCNQ4	PDZD7	WFS1	FOXI1	PTPRQ
DFNB31	KIAA1199	PMP22	MTTL1	ATP6V1B2	
DFNB59	LHFPL5	POU3F4	MTTI	PRPS1	
DIAPH1	LHX3	POU4F3	MTTQ	SMPX	
DSPP	LRTOMT	RDX	MTTM	COL9A2	
ERCC2	MARVELD2	SLC17A8	P2RX2	KCNQ1	
ERCC3	MTAP	SLC26A4	MTTS1	CEACAM16	
ESPN	LOXHD1	SLC26A5	MTTD	GIPC3	
ESRRB	MYH14	SLC4A11	MTTK	GPSM2	
EYA4	MYH9	SOX2	MTTH	ILDR1	

otogenetics corporation

Gene	NCBI Reference	OMIM	Description	CDS bps	# of Exons	Covered Region	AD*	AR*	X*	Syn. *
ACTB	NM_001101.3	102630	Actin β	1852	6	CDS only				√
ACTG1	NM_001199954.1	102560	Actin γ 1	2123	6	CDS only	√			
ATP6V1B1	NM_001692.3	192132	Lysosomal ATPase, H+ transporting, V1 subunit B1	1956	14	CDS only				√
ATP6V1B2	NM_001693.3	606939	Lysosomal ATPase, H+ transporting, V1 subunit B2	3054	14	CDS only				√
BCS1L	NM_001079866.1	603647	Ubiquinol-cytochrome c reductase synthesis-like	1454	8	CDS only				√
BSND	NM_057176.2	606412	Bartter syndrome, infantile, with sensorineural deafness	1396	4	CDS only		√		
CATSPER2	NM_001282310.1	607249	Cation channel, sperm associated 2	1755	13	CDS only				√
CCDC50	NM_178335.2	611051	Coiled-coil domain containing 50	8949	12	CDS only	√			
CDH23	NM_022124.5	605516	Cadherin-related 23	11134	70	CDS only		√		
CEACAM16	NM_001039213.2	614591	Carcinoembryonic antigen-related cell adhesion molecule 16	1692	7	CDS only	√			
CLDN14	NM_001146077.1	605608	Claudin 14	1469	3	CDS only		√		
CLRN1	NM_174878.2	606397	Clarin 1	2359	8	CDS only		√		
COCH	NM_001135058.1	603196	Cochlin	2882	11	CDS only	√			
COL11A2	NM_080680.2	120290	Collagen, type XI, α 2	6425	66	CDS only		√		
COL9A2	NM_001852.3	120260	Collagen, type IX, α 2	2831	32	CDS only				√
COL9A3	NM_001853.3	120270	Collagen, type IX, α 3	2485	32	CDS only				√
CRYM	NM_001888.3	123740	Crystallin, mu	1506	10	CDS only	√			
DFNA5	NM_004403.2	608798	Deafness, autosomal dominant 5	2521	10	CDS only	√			
DFNB31	NM_001173425.1	607928	Deafness, autosomal recessive 31 (Whirlin)	4076	12	CDS only		√		
DFNB59	NM_001042702.3	610219	Deafness, autosomal recessive 59	1534	7	CDS only		√		
DIAPH1	NM_005219.4	602121	Diaphanous-related formin 1	5804	28	CDS only	√			
DSPP	NM_014208.3	125485	Dentin sialophosphoprotein	4331	5	CDS only	√			
ECE1	NM_001397.2	600423	Endothelin converting enzyme 1	5114	19	CDS only				√
EDNRA	NM_001957.3	131243	Endothelin receptor type A	4168	8	CDS only				√
EDNRB	NM_000115.3	131244	Endothelin receptor type B	4296	8	CDS only				√
ERCC2	NM_000400.3	126340	Excision repair cross-complementing rodent repair deficiency, complementation group 2	2568	23	CDS only				√
ERCC3	NM_000122.1	133510	Excision repair cross-complementing rodent repair deficiency, complementation group 3	2751	15	CDS only				√
ESPN	NM_031475.2	606351	Espin	3531	13	CDS only		√		
ESRRB	NM_004452.3	602167	Estrogen-related receptor β	3029	11	CDS only		√		
EYA4	NM_004100.4	603550	Eyes absent homolog 4	5697	20	CDS only	√			
FAS	NM_000043.4	134637	Fas cell surface death receptor	2755	9	CDS only				√
FGF3	NM_005247.2	164950	Fibroblast growth factor 3	1548	3	CDS only				√
FGFR3	NM_000142.4	134934	Fibroblast growth factor receptor 3	4304	18	CDS only	√			
FOXI1	NM_012188.4	601093	Forkhead box I1	2296	2	CDS only		√		
GATA3	NM_001002295.1	131320	GATA binding protein 3	3070	6	CDS only				√
GIPC3	NM_133261.2	608792	GIPC PDZ domain containing family, member 3	4317	6	CDS only		√		
GJA1	NM_000165.3	121014	Gap junction protein, α 1	3130	2	CDS only		√		
GJB1	NM_000166.5	304040	Gap junction protein, β 1	1674	2	CDS only				√
GJB2	NM_004004.5	121011	Gap junction protein, β 2	2347	3	Entire gene + 5kb upstream		√		
GJB3	NM_001005752.1	603324	Gap junction protein, β 3	1777	2	CDS only	√			

GJB4	NM_153212.2	605425	Gap junction protein, β 4	2840	2	CDS only				√
GJB6	NM_001110219.2	604418	Gap junction protein, β 6	2178	5	CDS only	√			
GPR98	NM_032119.3	602851	G protein-coupled receptor 98	19333	90	CDS only		√		
GPSM2	NM_013296.4	609245	G-protein signaling modulator 2	3039	15	CDS only		√		
GRHL2	NM_024915.3	608576	Grainyhead-like 2	5231	16	CDS only	√			
GRXCR1	NM_001080476.2	613283	Glutaredoxin, cysteine rich 1	1003	4	CDS only		√		
GSTP1	NM_000852.3	134660	Glutathione S-transferase π 1	986	7	CDS only			√	
HAL	NM_002108.3	609457	Histidine ammonia-lyase	3927	21	CDS only				√
HGF	NM_000601.4	142409	Hepatocyte growth factor	2820	18	CDS only		√		
ILDR1	NM_001199799.1	609739	Immunoglobulin-like domain containing receptor 1	2908	8	CDS only		√		
JAG1	NM_000214.2	601920	Jagged 1	5988	26	CDS only				√
KCNE1	NM_000219.4	176261	Potassium voltage-gated channel, Isk-related family, member 1	3575	4	CDS only				√
KCNJ10	NM_002241.4	602208	Potassium inwardly-rectifying channel, subfamily J, member 10	5323	2	CDS only				√
KCNQ1	NM_000218.2	607542	Potassium voltage-gated channel, KQT-like subfamily, member 1	3262	16	CDS only				√
KCNQ4	NM_004700.3	603537	Potassium voltage-gated channel, KQT-like subfamily, member 4	4116	14	CDS only	√			
KIAA1199	NM_018689.1	608366	KIAA1199	7080	29	CDS only				√
LHFPL5	NM_182548.3	609427	Lipoma HMGIC fusion partner-like 5	2147	4	CDS only		√		
LHX3	NM_014564.3	600577	LIM homeobox 3	2419	6	CDS only				√
LOXHD1	NM_144612.6	613072	Lipoxygenase homology domains 1	6854	40	Chr18: 44152049-44152125		√		
LRTOMT	NM_001145309.3	612414	Leucine rich transmembrane and O-methyltransferase domain containing	3844	9	CDS only		√		
MARVELD2	NM_001038603.2	610572	MARVEL domain containing 2	2297	7	CDS only		√		
mir182	NR_029614.1	611607	microRNA 182	110	1	CDS only				√
mir183	NR_029615.1	611608	microRNA 183	110	1	CDS only				√
mir96	NR_029512.1	611606	microRNA 96	78	1	CDS only	√			
MITF	NM_198159.2	156845	Microphthalmia-associated transcription factor	4815	10	CDS only				√
MSRB3	NM_198080.3	613719	Methionine sulfoxide reductase B3	4307	6	CDS only		√		
MTAP	NM_002451.3	156540	Methylthioadenosine phosphorylase	4937	8	CDS only				√
MT-TD	NC_012920.1	590015	Mitochondrial transfer RNA aspartic acid	68	1	CDS only				√
MT-TH	NC_012920.1	590040	Mitochondrial transfer RNA histidine	69	1	CDS only				√
MT-TI	NC_012920.1	590045	Mitochondrial transfer RNA isoleucine	69	1	CDS only				√
MT-TK	NC_012920.1	590060	Mitochondrial transfer RNA lysine	70	1	CDS only				√
MT-TL1	NC_012920.1	590050	Mitochondrial transfer RNA leucine	75	1	CDS only				√
MT-L2	NC_012920.1	590055	Mitochondrial transfer RNA leucine2	71	1	CDS only				√
MT-TM	NC_012920.1	590065	Mitochondrial transfer RNA methionine	68	1	CDS only				√
MT-TQ	NC_012920.1	590030	Mitochondrial transfer RNA glutamine	72	1	CDS only				√
MT-TS1	NC_012920.1	590080	Mitochondrial transfer RNA serine 1	69	1	CDS only				√
MT-TS2	NC_012920.1	590085	Mitochondrial transfer RNA serine 2	59	1	CDS only				√
MYH14	NM_001145809.1	608568	Myosin, heavy chain 14, non-muscle	6930	43	CDS only	√			
MYH9	NM_002473.4	160775	Myosin, heavy chain 9, non-muscle	7505	41	CDS only	√			
MYO15A	NM_016239.3	602666	Myosin XVA	11876	66	CDS only		√		
MYO1A	NM_001256041.1	601478	Myosin IA	3658	29	CDS only	√			
MYO1C	NM_001080779.1	606538	Myosin IC	4973	32	CDS only				√
MYO1F	NM_012335.3	601480	Myosin IF	4173	28	CDS only				√

MYO3A	NM_017433.4	606808	Myosin IIIA	5798	35	CDS only		√		
MYO6	NM_004999.3	600970	Myosin IV	8662	35	CDS only		√		
MYO7A	NM_000260.3	276903	Myosin 7A	7465	49	CDS only		√		
NDP	NM_000266.3	300658	Norrie disease	2058	3	CDS only				√
NR2F1	XM_005272070.1	132890	Nuclear receptor subfamily 2, group F, member 1	1983	ND	CDS only				√
OTOA	NM_144672.3	607038	Otoancorin	3624	28	CDS only		√		
OTOF	NM_194248.2	603681	Otoferlin	7171	47	CDS only		√		
OTOR	NM_020157.3	606067	Otoraplin	1482	4	CDS only				√
P2RX2	NM_170683.3	600844	Purinergic receptor P2X, ligand-gated ion channel, 2	1945	10	CDS only	√			
PAX3	NM_181457.3	606597	Paired box 3	2032	8	CDS only				√
PCDH15	NM_001142763.1	605514	Protocadherin-related 15	7042	33	CDS only		√		
PDZD7	NM_001195263.1	612971	PDZ domain-containing 7	4164	17	CDS only				√
PMP22	NM_000304.3	601097	Peripheral myelin protein 22	1861	5	CDS only				√
POU3F4	NM_000307.4	300039	POU class 3 homeobox 4	1507	1	CDS only			√	
POU4F3	NM_002700.2	602460	POU class 4 homeobox 3	1182	2	CDS only	√			
PRPS1	NM_002764.3	311850	Phosphoribosyl pyrophosphate synthetase 1	2156	7	CDS only			√	
PTPRQ	NM_001145026.1	603317	Protein tyrosine phosphatase, receptor type, Q	8066	3	Chr12: 80849273-80849842; 80878212-80878383		√		
RDX	NM_001260492.1	179410	Radixin	2761	14	CDS only		√		
SERPINB6	NM_004568.5	173321	Serpin peptidase inhibitor, clade B (ovalbumin), member 6	1932	7	CDS only		√		
SIX1	NM_005982.3	601205	SIX homeobox 1	2687	2	CDS only	√			
SIX5	NM_175875.4	600963	SIX homeobox 5	3352	3	CDS only				√
SLC17A8	NM_139319.2	607557	Solute carrier family 17 (vesicular glutamate transporter), member 8	3983	12	CDS only	√			
SLC26A4	NM_000441.1	605646	Solute carrier family 26 (anion exchanger), member 4	4930	21	CDS only		√		
SLC26A5	NM_198999.2	604943	Solute carrier family 26 (anion exchanger), member 5	2697	20	CDS only		√		
SLC4A11	NM_001174090.1	610206	Solute carrier family 4, sodium borate transporter, member 11	3268	20	CDS only				√
SMPX	NM_014332.2	300226	Small muscle protein, X-linked	951	5	CDS only			√	
SNAI2	NM_003068.4	602150	Snail family zinc finger 2	2112	3	CDS only				√
SOX2	NM_003106.3	184429	SRY (sex determining region Y)-box 2	2520	1	CDS only				√
SPINK5	NM_001127698.1	605010	Serine peptidase inhibitor, Kazal type 5	3745	34	CDS only				√
STRC	NM_153700.2	606440	Stereocilin	5515	29	CDS only		√		
TBL1X	NM_005647.3	300196	Transducin (β)-like 1X-linked	5715	18	CDS only				√
TCF21	NM_198392.2	603306	Transcription factor 21	3249	3	CDS only				√
TECTA	NM_005422.2	602574	Tectorin α	6468	23	CDS only		√		
TFCP2	NM_005653.4	189889	Transcription factor CP2	3715	15	CDS only				√
TIMM8A	NM_004085.3	300356	Translocase of inner mitochondrial membrane 8 homolog A	1459	2	CDS only			√	
TJP2	NM_004817.3	607709	Tight junction protein 2	4725	23	CDS only	√			
TMC1	NM_138691.2	606706	Transmembrane channel-like 1	3201	24	CDS only		√		
TMIE	NM_147196.2	607237	Transmembrane inner ear	1861	4	CDS only		√		
TMPRSS3	NM_024022.2	605511	Transmembrane protease, serine 3	2463	13	CDS only		√		
TMPRSS5	NM_030770.2	606751	Transmembrane protease, serine 5	2233	13	CDS only				√
TPRN	NM_001128228.2	613354	Taperin	2641	4	CDS only		√		

TRIOBP	NM_001039141.2	609761	TRIO and F-actin binding protein	10159	24	CDS only		√
USH1C	NM_153676.3	605242	Usher syndrom 1C	3246	28	CDS only		√
USH1G	NM_173477.3	607696	Usher syndrome 1G	3568	3	CDS only		√
USH2A	NM_206933.2	608400	Usher syndrome 2A	18883	72	CDS only		√
WFS1	NM_006005.3	606201	Wolfram syndrome 1	3640	8	CDS only	√	

* Inheritance pattern: AD = autosomal dominant
AR = autosomal recessive
X = X-linked
Syn = syndromic deafness
